# Reconstructing traits, not restoring lineages: the biological limits of de-extinction

**DOI:** 10.1098/rsbl.2026.0315

**Published:** 2026-11-11

**Authors:** Andrew Davinack, Charlotte K. Daniels

**Affiliations:** Department of Biological, Chemical, and Environmental Sciences, Wheaton College Massachusetts, Norton, MA, USA

**Keywords:** genomic, reconstruction, conservation, genetics

## Abstract

Recent advances in ancient DNA sequencing, comparative genomics and genome editing have renewed interest in de-extinction, often framed as the technological reversal of species loss. However, this framing risks conflating the reconstruction of selected genomic or phenotypic traits with the recovery of extinct species themselves. Here, we argue that de-extinction cannot recover extinct species because extinction represents the termination of an evolutionary lineage, not just the disappearance of genetic information. Species are historically continuous population-level lineages shaped by gene flow, mutation, drift, selection, demographic history and ecological interaction. A reconstructed genome, even if highly accurate, represents at most a partial snapshot of an individual organism; it cannot reproduce the population structure, haplotypic diversity, demographic trajectory or historical ecological context associated with the extinct lineage. We label this error as lineage–genome conflation: the treatment of genomic reconstruction as equivalent to lineage recovery. Contemporary de-extinction approaches therefore produce engineered organisms derived from extant lineages that incorporate selected variants or traits inferred from extinct taxa. Such organisms may have scientific or conservation value as analogues or ecological proxies, but they are not continuations of extinct lineages.

## 1. Introduction

Recent advances in genome sequencing, ancient DNA analysis and gene editing have renewed scientific and public interest in ‘de-extinction’, broadly framed as the reconstruction of extinct organisms through the genetic modification of extant relatives [1–3]. These efforts are often presented as a technological reversal of extinction, suggesting that sufficiently advanced genomic manipulation could restore species that have disappeared from the Earth. This narrative has gained prominence through high-profile announcements and widespread media coverage.

A notable example was a recent claim that genetically engineered grey wolves (*Canis lupus*) constitute, or approximate, the de-extinction of the extinct dire wolf (*Aenocyon dirus*), an apex predator that disappeared near the end of the Pleistocene. Dire wolves represented a deeply divergent canid lineage that separated from the ancestors of living wolf-like canids approximately 5.7 million years ago and showed no evidence of recent introgression with grey wolves, coyotes or their common ancestor [4]. According to the company's publicly reported methodology, ancient dire-wolf sequences were compared with extant canid genomes, after which 20 targeted edits across 14 genes were introduced into grey-wolf cells. Nuclei from the edited cells were then used to generate cloned animals via surrogate hosts [5]. Because ancient DNA is inherently fragmentary and unevenly preserved [6], complete genome reconstruction—and, more importantly, the recovery of complete population-level genomic diversity—is not feasible. As a result, these approaches rely on modifying a limited number of loci within the genome of a closely related extant species. The resulting organisms, while viable, remain derived from the grey wolf lineage.

Although the scientific literature commonly distinguishes among ‘proxies’, ‘analogues’ and ‘functional replacements’, commercial and popular-media accounts have sometimes presented engineered organisms as successful restorations of extinct species. Colossal Biosciences, for example, described its genetically engineered canids as the successful restoration and revival of the dire wolf, terminology subsequently reproduced in prominent media coverage [5,7]. Our criticism here refers primarily to the commercial and public-facing claims of literal species revival, rather than to the more qualified terminology generally employed in peer-reviewed scientific and conservation literature. This framing nevertheless raises a deeper conceptual issue: while genomic engineering may reproduce selected traits inferred from extinct organisms, it does not necessarily restore the historical lineage from which those traits were derived. Species correspond to evolving lineages characterized by population continuity, genetic variation and ecological context [8–10]. Here, we argue that claims of de-extinction require a lineage-based evaluation of species identity: genomic reconstruction may recreate selected traits or produce ecological proxies, but it cannot recover extinct species as historically continuous evolutionary lineages.

## 2. Technological pathways to de-extinction

The term ‘de-extinction’ encompasses several technological pathways that differ substantially in ancestry, genomic fidelity, developmental origin and their relationship to the extinct population. These approaches should not be treated as conceptually equivalent (figure 1).

**Figure 1.**
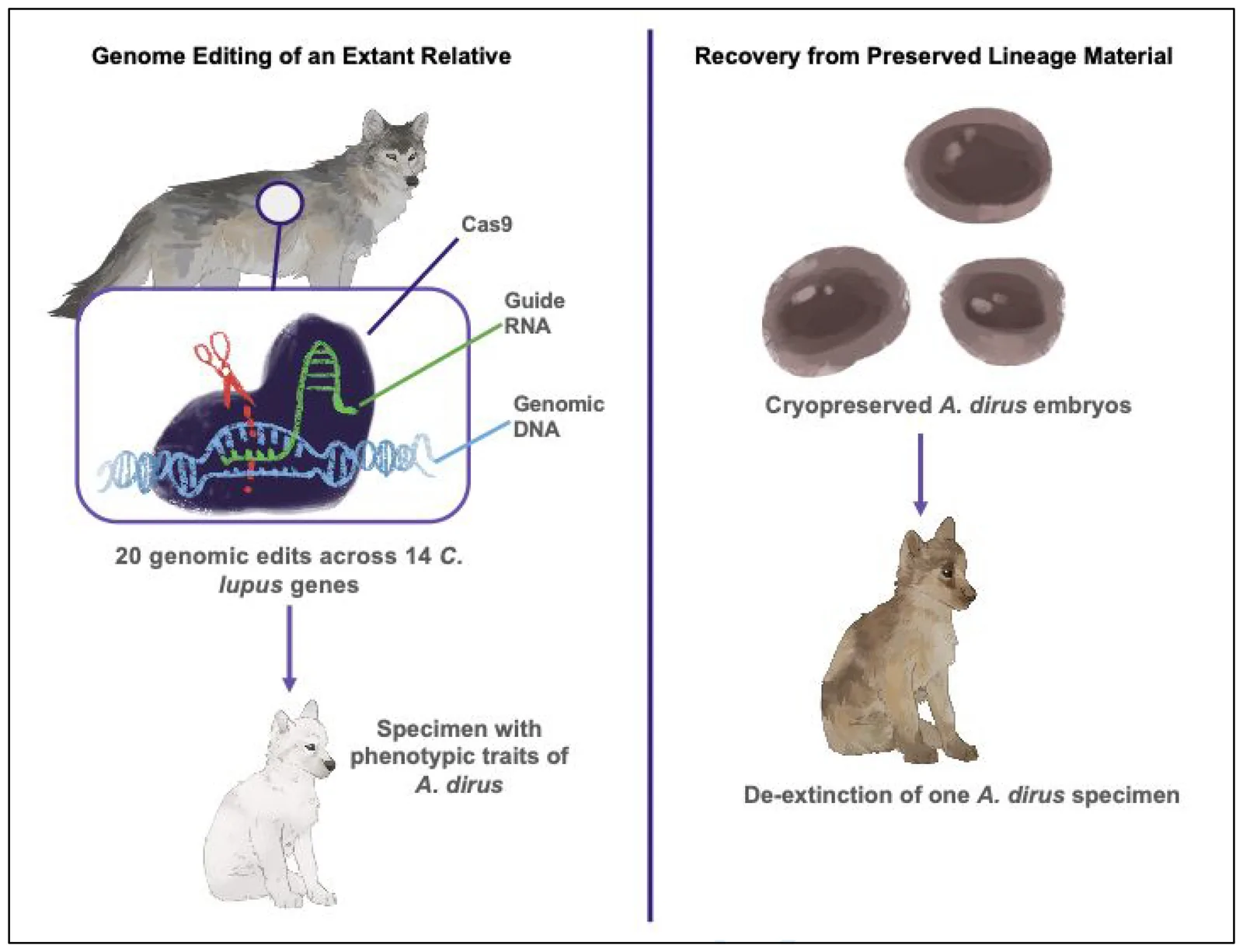
Contrasting biological pathways commonly grouped under ‘de-extinction’. Left: comparative-genomic editing introduces selected variants associated with an extinct taxon into cells of an extant relative. Right: recovery from a hypothetical cryopreserved *A. dirus* embryo represents a fundamentally different pathway because the embryo is preserved biological material produced by the historical lineage itself.

Selective back-breeding attempts to recover ancestral phenotypes by breeding extant descendants or relatives that retain subsets of the relevant traits [1,2,11]. Although such populations may approximate aspects of the extinct phenotype, they remain genealogically continuous with the living populations from which they were bred. Genome-editing approaches similarly introduce variants inferred from extinct taxa into an extant relative. The resulting organisms are derived developmentally and genealogically from the extant lineage, even when they express traits associated with the extinct taxon.

Cloning from preserved viable cells presents a different case [12]. An organism produced from an intact somatic cell of a recently extinct individual retains direct biological and genealogical connection to the historical population, although the resulting population may contain only a small fraction of the original species' genetic variation. Recovery from cryopreserved embryos represents an even stronger case of biological continuity because the embryo is an existing developmental stage produced by the historical population rather than a genome reconstructed after extinction.

Germline-based approaches may transmit genetic material from an endangered or extinct lineage through a surrogate reproductive system, whereas synthetic-genomic approaches seek to reconstruct larger portions of extinct genomes from preserved sequence information [2]. Where numerous well-preserved historical specimens are available, genomic sampling may recover substantially more of the former species' genetic variation and population structure than approaches based on a single specimen or a limited set of trait-associated variants [13].

Our central argument therefore applies most directly to comparative-genomic and genome-editing approaches in which selected extinct-associated variants are introduced into an extant lineage. Other pathways, particularly cloning and embryo recovery raise more difficult questions about biological continuity and should be evaluated separately. Distinguishing these pathways prevents conclusions drawn from current trait-engineering projects from being generalized uncritically to all conceivable forms of de-extinction. These technological distinctions matter because each pathway raises a different question about what, biologically, would count as restoration of a species.

## 3. Species concepts and lineage restoration

Clarifying the limits of de-extinction requires distinguishing among the different biological properties used to define and recognize species. Species concepts remain plural and contested, and different frameworks emphasize reproductive cohesion, diagnosability, ecological distinctiveness, phenotype or historical descent to different degrees [8–10,14,15]. These approaches do not necessarily produce identical classifications in every case. Nevertheless, many contemporary species concepts treat species as historically extended biological entities rather than as fixed collections of genes or traits.

The biological species concept defines species in terms of reproductive connectivity, emphasizing gene flow within populations and reproductive isolation from others [16]. The phylogenetic species concept identifies species as diagnosable groups reflecting shared ancestry [17]. The evolutionary species concept places greater emphasis on temporal continuity, defining species as lineages with their own evolutionary tendencies and historical fate [8]. Ecological species concepts, by contrast, emphasize the occupation of a distinct niche or adaptive zone [18]. Under a strongly ecological interpretation, an engineered population that expressed the relevant adaptations and performed a comparable ecological role might plausibly be regarded as a functional or ecological replacement for an extinct species.

Such ecological, phenotypic or diagnostic equivalence, however, is not identical to restoration of the historical lineage itself. Similar traits or ecological functions can arise through convergence, selective breeding or genetic engineering and therefore do not independently establish descent from the extinct population. The general lineage concept provides one framework for separating these issues by treating species as segments of separately evolving population-level lineages, while reproductive isolation, diagnosability, ecology and phenotype serve as evidence used to recognize those entities [14,15]. From this perspective, an organism may resemble an extinct species or occupy a similar niche without constituting a continuation of its evolutionary history.

This distinction has direct implications for de-extinction. Historical genomes may recover aspects of phenotype, genetic variation or former population structure, but genomic information alone does not re-establish the genealogical continuity of an extinct species. Treating genomic reconstruction as sufficient for species restoration therefore risks reducing species identity to genomic composition; a form genetic essentialism [19,20].

Species recognition proceeds through abductive inference. When biologists use the term ‘species delimitation’ what we are really discussing are explanatory hypotheses evaluated according to how well they account for observed genetic, ecological, morphological, reproductive and historical evidence [21,22]. Our argument does not require that all species concepts impose an identical criterion of uninterrupted continuity. Rather, it concerns the stronger claim that the extinct species itself has been restored. Reconstructing traits, ecological function or diagnostic features may justify describing an organism as an analogue, proxy or functional replacement, but such outcomes should be distinguished from restoration of the historical lineage that became extinct.

A lineage-based account also does not imply that species cease to exist whenever their populations become small, enter captivity or encounter novel ecological conditions. A final surviving individual remains an authentic member of its species because it is genealogically continuous with the historical population from which it descended. Similarly, a translocated population does not lose its species identity simply because it occupies a different ecological community. Population collapse and ecological displacement may reduce evolutionary potential or eliminate ecological function, but they do not erase ancestry. Contemporary genome-editing approaches present a different case however. An organism produced by modifying an extant relative originates developmentally and genealogically within the extant lineage, even when it expresses traits inferred for an extinct taxon. The biological distinction in contemporary genome-editing projects is therefore between continuity with the historical lineage and construction within a different one.

More difficult boundary cases arise however, when viable biological material from the historical population has been preserved. An organism produced from a somatic cell collected from the final living individual, or development from a cryopreserved embryo, retains a more direct biological and genealogical connection to the historical population than an organism produced by editing an extant relative. Cryopreserved embryos, in particular, may be understood as preserved developmental stages of the lineage rather than genomic reconstructions created after its termination. Such cases therefore resemble delayed reproduction or recovery from preserved lineage material and should not be treated as conceptually equivalent to trait engineering in a related species. By contrast, synthesizing one or several genomes inferred from ancient or historical specimens could recover a greater proportion of former genetic variation, but the resulting population would still be newly assembled from preserved information rather than developed from viable biological material continuous with the historical population. The boundary between lineage persistence and extinction may therefore be difficult to define when viable cells or embryos remain, but this ambiguity does not apply in the same way to contemporary approaches that edit selected variants into an extant lineage, as in the case of the dire wolf case study.

## 4. Population genetics and the loss of lineage diversity

Population genetics provides a mechanistic explanation for why genomes cannot represent species. Genetic variation is distributed across individuals and populations, and evolutionary processes act on this variation over time [23]. Consequently, a reconstructed genome, or even a panel of historical genomes, can recover only part of the genetic variation and population structure once present in an extinct lineage.

Natural populations exhibit standing genetic variation shaped by mutation, gene flow, genetic drift and selection. These processes operate across populations and generations, producing patterns of diversity that emerge only at the population level [24]. For this reason, the evolutionary properties of a species cannot be inferred from a single individual genome.

Population structure further limits the representativeness of any single genome. Many species are subdivided into partially isolated populations that differ in allele frequencies as a result of restricted gene flow, geographic barriers, environmental heterogeneity, drift and local demographic history [25,26]. Phylogeographic processes—including range expansion, contraction, and secondary contact—generate complex spatial patterns of genetic differentiation that cannot be reduced to a single genotype [27]. Haplotypic variation illustrates this point. Haplotypes capture patterns of ancestry and connectivity among populations and reflect the historical processes that shape lineages [27]. These patterns may sometimes be inferred from sufficiently broad historical sampling, but they cannot be fully restored merely by assembling one or more engineered genomes. Demographic history adds further complexity. Events such as bottlenecks, expansions and migrations leave signatures in population-level genetic variation, allowing researchers to infer aspects of a lineage's trajectory through time [28,29]. These signatures cannot be reproduced through genomic reconstruction, which relies on limited and temporally constrained data.

Together, these considerations show that individual genomes cannot capture the former population-level diversity, structure and demographic history of an extinct lineage. Even when historical sampling permits aspects of these properties to be inferred, assembling one or more genomes does not restore the population processes through which they arose. These population-genetic limitations become especially important when reconstructed traits are interpreted as evidence that an extinct lineage itself has been restored.

## 5. Lineage–genome conflation in de-extinction claims

The preceding distinctions expose what we term *lineage–genome conflation*: treating the reconstruction of selected genomic features as equivalent to recovering the evolutionary lineage from which those features were inferred. This conflation occurs when extinct-associated variants introduced into a living surrogate are interpreted as establishing the revival or continuation of the extinct species itself. It is most evident in public-facing claims that move from the recreation of particular traits to the stronger assertion that the historical species has been restored.

This distinction is particularly clear in the recent dire-wolf case. The project did not begin with viable dire-wolf cells, embryos or other preserved biological material continuous with the extinct population. Instead, ancient dire-wolf sequence data were compared with genomes from living canids to identify variants associated with selected inferred traits. A subset of these variants was then introduced into grey-wolf cells, which were used to produce cloned animals [5]. Thus, the procedure represents comparative-genomic trait engineering within an extant lineage rather than cloning or propagation of preserved dire-wolf material.

The resulting animals remain genealogically, cellularly and developmentally derived from the grey-wolf lineage. Introducing variants associated with inferred dire-wolf traits may alter aspects of morphology, physiology or appearance, but it does not establish descent from *Aenocyon dirus*, reconstruct the complete dire-wolf genome or recover the geographic population structure and genetic variation formerly distributed across that species. The animals therefore display selected dire-wolf-associated characteristics while remaining novel engineered members of an extant canid lineage.

The dire-wolf case therefore illustrates lineage–genome conflation in its clearest form: selected traits inferred from an extinct lineage were reconstructed within the cellular, developmental and genealogical context of an extant one. The result may be a scientifically informative analogue, but the intervention does not establish continuity with *Aenocyon dirus*.

## 6. Ecological context and the irreversibility of extinction

Ecological context presents a different limitation from lineage discontinuity. Species persist through ecological change, and an organism does not cease to belong to a species simply because it inhabits a novel environment or has lost some historical interactions. Nevertheless, de-extinction cannot restore the historical ecological system in which an extinct lineage evolved. An engineered dire-wolf analogue would enter a contemporary ecological network whose prey assemblages, competitors, parasites, climate and selective conditions differ from those of Late Pleistocene North America. It might perform some functions associated with the extinct taxon, but this would constitute functional substitution under contemporary conditions rather than restoration of the original evolutionary-ecological relationship. Claims of de-extinction should therefore specify what form of restoration is being proposed. Restoration of a historical lineage concerns genealogical continuity with the extinct population. Restoration of former genetic diversity concerns the recovery or reintroduction of historical variants and population structure. Phenotypic restoration concerns the recreation of morphological, physiological or behavioural traits. Ecological-function restoration concerns whether an organism performs roles comparable to those of the extinct taxon under contemporary conditions. Restoration of the historical ecological community would require recovery of the original network of species interactions and environmental conditions. Success along one of these dimensions does not necessarily imply success along the others. These distinctions have practical consequences because lineage continuity, genetic recovery, phenotypic reconstruction, ecological substitution and historical community restoration require different evidence, imply different conservation goals and should not be evaluated using the same criteria of success.

## 7. From de-extinction to conservation genomics

Among the pathways described above, comparative-genomic and gene-editing projects are best evaluated as engineered analogues, ecological proxies or phenotypic approximations rather than as restorations of extinct lineages [30]. Their scientific value may lie in testing genotype–phenotype relationships, evaluating functional effects of extinct-associated variants or investigating whether engineered organisms can perform particular ecological roles under contemporary conditions. Framing success in these terms avoids conflating trait construction with literal species revival and provides clearer criteria for evaluating both the potential and limitations of these technologies.

Interestingly, these same technologies may have greater conservation value when applied to extant species. Historical genomes preserved in museum collections could reveal alleles and population structure lost through recent bottlenecks, while genomic-assisted breeding or gene editing may allow some of that variation to be reintroduced into living populations. Such applications do not reverse extinctions of course, but they may slow genomic erosion, reduce inbreeding depression and restore adaptative potential before lineage termination occurs. In this sense, the most consequential legacy of de-extinction research may lie not in resurrecting extinct taxa, but in strengthening the evolutionary resilience of species that still survive.

## 8. Conclusion: extinction as lineage termination

Extinction terminates a historical evolutionary lineage, but the technologies grouped under de-extinction differ in what they can recover. Viable cells or cryopreserved embryos may preserve forms of biological continuity, whereas comparative-genomic editing reconstructs selected traits within an extant lineage. Historical genomes may recover information about former variation, phenotype or population structure, but genomic information alone does not re-establish the demographic and genealogical history through which the extinct lineage persisted.

Greater precision in terminology therefore has practical consequences. Lineage restoration, genetic recovery, phenotypic reconstruction, ecological substitution and restoration of historical communities are distinct objectives and require different criteria of success. We argue here that the most immediate conservation value of these technologies lies not in claims of reversing extinction, but in preserving genetic variation, ecological interactions and evolutionary potential in species that are still alive.

## Data Availability

This article has no additional data.
